# Targeting potential receptor molecules in non-small cell lung cancer (NSCLC) using in silico approaches

**DOI:** 10.3389/fmolb.2023.1124563

**Published:** 2023-02-09

**Authors:** C. Kirubhanand, J. Merciline Leonora, S. Anitha, R. Sangeetha, K. T. Nachammai, K. Langeswaran, S. Gowtham Kumar

**Affiliations:** ^1^ Department of Anatomy, All India Institute of Medical Sciences, Nagpur, Maharashtra, India; ^2^ PG and Research Department of Physics, Government Arts College, Madurai, Tamil Nadu, India; ^3^ Department of Physics, ArulmiguPalaniandavar College of Arts and Science, Palani, Tamil Nadu, India; ^4^ Department of Physics, Mannar Thirumalai Naicker College, Madurai, Tamil Nadu, India; ^5^ Department of Biotechnology, Science Campus, Alagappa University, Karaikudi, Tamil Nadu, India; ^6^ Faculty of Allied Health Sciences, Chettinad Hospital & Research Institute, Chettinad Academy of Research and Education, Kelambakkam, Tamil Nadu, India

**Keywords:** lochnericine, quantum chemical calculations, molecular docking, molecular dynamics, anticancer study, non-small lung cancer

## Abstract

**Introduction:** Non-Small Cell Lung Cancer is the most prevalent type of cancer in lung cancer. Chemotherapy, radiation therapy, and other conventional cancer treatments have a low success rate. Thus, creating new medications is essential to halt the spread of lung cancer.

**Methods:** In this study bioactive nature of lochnericine against Non-Small Cell Lung Cancer (NSCLC) was analyzed using various computational approaches such as quantum chemical calculations, molecular docking, and molecular dynamic simulation. Furthermore, the MTT assay shows the anti-proliferation activity of lochnericine.

**Results and Discussion:** Using Frontier Molecular Orbital (FMO), the calculated band gap energy value associated with bioactive compounds and the molecule’s potential bioactivity is confirmed. The H38 hydrogen atom and O1 oxygen atom in the molecule are effectively electrophilic, and potential nucleophilic attack sites were confirmed through analysis of the Molecular electrostatic potential surface. Furthermore, the electrons within the molecule were delocalized, which confers bioactivity on the title molecule and was authorized through Mulliken atomic charge distribution analysis. A molecular docking study revealed that lochnericine inhibits non-small cell lung cancer-associated targeted protein. The lead molecule and targeted protein complex were stable during molecular dynamics simulation studies till the simulation period. Further, lochnericine demonstrated remarkable anti-proliferative and apoptotic features against A549 lung cancer cells. The current investigation powerfully suggests that lochnericine is a potential candidate for lung cancer.

## 1 Introduction

Developed and developing nations consider cancer a significanthealth concern with increasing incidence and mortality rates. The tumor is caused by complex biological processes, namely uninhibited cell proliferation, cell death resistance, neo-angiogenesis, and metastasis (Hausman, 2019). Incursion and metastasis are the frequent causes of cancer-related mortality, resulting in additional invasion sites and severe organ damage. Cases of cancer are rapidly becoming a risk factor for various malignancies, most notably lung, liver, colorectal, and breast cancers. Because of the disease’s high notoriety, therapy has been a never-ending fight with few results (Mullard, 2020; Banaganapalli et al., 2022). One of the deadliest and most ubiquitous forms of cancer in people is lung cancer. The most prevalent type of cancer is NSCLC (non-small cell lung cancer), estimated as 85% among other cancers. Nearly 90% of non-small cell lung cancer occurrences are caused by smoking. Tumor-infiltrating lymphocytes (TILs), in general, and notably CD8^+^ TILs, are associated with a favourable prognosis in non-small cell lung cancer (NSCLC) (Gueguen et al., 2021). Traditional cancer treatments, such as surgery, radiation, and chemotherapy, have a low success rate. Thus, developing new drugs is crucial to stop lung cancer spread (Schabath and Cote, 2019).

In cancer treatment, natural products may be used in addition to traditional anticancer medications. Recently, researchers worldwide have focused on developing novel drugs with medicinal potential derived from natural sources such as plants. Plants and their phytoconstituents have been used for medical purposes since antiquity (Ekiert and Szopa, 2020). Natural-source chemicals attract the interest of scientists worldwide. Humans have extracted cancer chemotherapeutic chemicals from flora (Deng et al., 2020). Lochnericine is a significant monoterpene indole alkaloid (MIA), found in the roots of Madagascar periwinkle (*Catharanthusroseus*) and formed by the stereoselective C6, C7-epoxidation of tabersonine. The stereoselective C6 produces Lochnericine and C7-epoxidation of tabersonine and can be subsequently metabolized to produce more complex MIAs. Although the enzymes in charge of its downstream modifications have been identified, those in charge of lochnericine production have not.

Growth factors, namely hormones, mainly influence the migration of normal cells and the division of cells. Tyrosine kinase EGRF (Epidermal Growth Factor Receptor) influences cell proliferation, cell division, and tumour development. It is a representative transmembrane receptor which triggers signalling pathways *via* ligand-provoked dimerization (Kavitha et al., 2015; Zhou and Yao, 2016; Shaik et al., 2019). In human cancers, the most prevalently mutated oncogene is the Kirsten rat sarcoma (KRAS) gene involved in carcinogenesis, which accounts for more than twenty per cent of lung cancer, particularly NSCLC (Reck et al., 2021). There is a strong connotation between various solid tumours NTRK (Neurotrophic Tropomyosin Receptor Kinase) gene fusion, including NSCLC. In cancers covering NSCLC, ALK activation occurs through fusion gene formation, the preliminary actuating mutation in ALK. TRIM 1 is designated by cancer cells to encourage tumorigenic development and is upregulated in malignant cells. In tumour cells, it indorses elevatedproteasome activity (Wang et al., 2016). This study investigated a lochnericine molecule using DFT quantum chemical calculations, including molecular structure, vibrational (FMOs), and other vibrational analyses. The computational studieslikely docking analysis was also performedusing bioinformatics tools to check the molecule’s repressivebehaviour against lung cancer-associated targeted proteins. Computer-Aided Drug Design (CADD) has emerged as an effective method for determining potential lead compounds and improving the development of new treatments for various diseases. Molecular dynamics simulations determine the stability of protein-ligand complexes. Furthermore, *in vitro* cytotoxicity testing was carried out to confirm the anti-lung cancer activity.

## 2 Materials and methods

### 2.1 Quantum chemical calculations

The most stable molecular structure of lochnericine was optimized using the Gaussian 09 software, and the DFT/B3LYP method with LANL2DZ was used to optimize the structure. The Los Alamos National Laboratory 2 double zeta (LANL2DZ) for transition metals and all-electron basis sets for all other non-transition metal atoms are used more frequently in computations on systems including transition metals. (Frisch et al., 2009). The molecule’s vibrational wavenumbers were computed and assigned using the VEDA 4.0 software (Jamroz, 2004). The optimized molecular structure, vibrational wavenumbers, FMOs, MEP surface, and Mullikenatomic charge distributions of Lochnericine molecule were visualized using GaussView 05 software (Dennington et al., 2009). All the quantum chemical calculations were performed without regard for the potential energy surface to grasp the interception nature of the Lochnericine molecule.

### 2.2 Bioinformatics study

#### 2.2.1 Target and ligand preparation

In the present study, target receptor molecules, including EGFR [PDB ID: 2ITY], KRAS [PDB ID: 7LGI], Ntrk [PDB ID: 7VKO], and ALK [PDB ID: 2XP2], were obtained from the RCSB Protein Data Bank (Sussman et al., 1998). TRIM11 protein sequences were obtained from UniProt (UniProt ID: Q96F44) (UniProt, 2019). The 3D protein structure of the other protein, TRIM11, was modelled using the Swiss model (Schwede et al., 2003). Procheck in the SAVES server was used to verify the three-dimensional protein structure (Laskowski et al., 2016). All water molecules and irregular residues were purified from the primary protein structure. In the PyRx workspace folders, the protein receptor was saved as a PDBQT file after adding missing hydrogens and charges. Using the PubChem database, the lochnericine molecule was downloaded. The ligand was imported and prepared. These ligands were then converted to Auto Dock Ligand format (PDBQT).

#### 2.2.2 Molecular docking

Molecular docking was performed using Vina version 2.0 in PyRX (Dallakyan and Olson, 2015). In the PyRX interface, rigid docking was carried out for the targeted receptor molecules. The ligand and proteins were then subjected to docking to get the binding affinity with each other. Lamarckian genetic algorithm conformational search with the default parameters was utilized. Further, the grid on the targeted protein’s ligand-binding site was situated in the centre of the binding site. Protein-lead molecule interactions were analyzed using Discovery Studio Visualize software.

#### 2.2.3 ADME prediction

ADME prediction was performed in the SWISS ADME tool (Tripathi et al., 2019). This tool can forecast the pharmacokinetics and drug-like characteristics of active compounds. The numbers of rotatable bonds, hydrogen bond donors, acceptors, and molecular weight, were also predicted. The log *p* values represent the lipophilicity of the compounds. The logarithmic S value represents the solubility of water. Human gastrointestinal absorption (HIA) and blood-brain barrier (BBB) permeation were predicted pharmacokinetic properties in swiss adme.

#### 2.2.4 Molecular dynamics simulation

In simulation studies using molecular dynamics (MD), the atomic and molecular motions within the protein structure were examined. Atoms and molecules can interact for 100 ns in the GROMACS (GROningenMAchine for Chemical Simulations) (Van Der Spoel et al., 2005). Macromolecular structure-to-function interactions may now be efficiently understood using an advanced technology known as molecular dynamics simulations (Awan et al., 2021; Bima et al., 2022). MD simulations were performed by using the GROMOS96 43a1 force field. Targeted receptor molecule and ligand complex topology was created by parametrizing the compound through the PRODRG server (Schüttelkopf and Van Aalten, 2004; Sangavi and Langeswaran, 2021). The simple point charge (SPC216) water model’s step for salvation is the cubic box centre to edges. To balance the MD simulations, counter ions (Na^+^ and Cl^−^) were also included in the simulation systems (Shaik et al., 2022). Adding counter-ions to the system to neutralize it. Two methods exist for adding ions: Solve the solution and then add ions to replace the solvent’s randomly distributed molecules. To solve a problem, distribute ions in accordance with the macromolecule’s electrostatic potential. The simulation box interacts with an endless number of its periodic images when periodic boundary conditions are utilized, and the Coulomb energy is calculated using grid-based techniques. As a result, if a simulation system is charged, the electrostatic energy will accumulate to infinity. We must neutralize the system by introducing counter-ions to address this problem and allow simulations to determine the electrostatic energy accurately. Using MD simulations at the atomistic level has the advantage of allowing for capturing and evaluating various motional contributions to the overall complex dynamics of the targeted receptor molecule. Potential steric obstacles between the atoms in the solvated system were removed using the energy minimization step and the 1,500 steepest descent followed by conjugate gradient techniques. The NVT and NPT ensemble’s two-step equilibration was carried out for 100 ps at constant volume, with gradual heating from 0 to 300 K, and at a pressure of 1 atm. The temperature is maintained with a Berendsen thermostat. The Parrinello-Rahman barostat is used to keep the system’s pressure constant. The simulation of each system took place for 100 ns? Xmgrace software was used to examine the flexibility and stability of the protein and ligand complex (Turner, 2005).

### 2.3 Cell line studies

#### 2.3.1 Materials and drug preparation

Sigma, St Louis, MO, United States, supplied the dimethyl sulfoxide (DMSO) and Lochnericine. All other chemicals and solvents were purchased from SISCO Research Laboratories (SRL). Lochnericine was synthesized in a serum-free RPMI medium after being dissolved in dimethyl sulfoxide (DMSO) at a final concentration of less than 0.1 percent (v/v). It did not affect cell viability) and filtered through a 0.045 mm syringe filter before being stored at 4°C.

#### 2.3.2 Cell proliferation assay by MTT method

The MTT assay was employed to assess the anti-proliferative efficacy of lochnericine against A549 lung cancer cells. Through mitochondrial enzymes, the translation of MTT (3-(4,5-dimethylthiazol-2-yl)-2,5-diphenyltetrazolium bromide) to the formation of MTT is the critical mechanism involved in this cytotoxicity assay. NCCS Pune supplied the lung cancer cell line A549 for this investigation. In 24 well tissue culture plates, 2x 10^5^ cells were cultured with 0.5 mL medium/well, 37°C temperature was maintained with CO_2_ 5%, and relative humidity was about 95%. For 48 h, cells were treated with various concentrations of lochnericine. The cells were incubated at 37°C for 4 h after adding 200 µl of MTT solution (5 mg/mL), and cells were harvested. Through the gentle aspiration method, the medium and unread MTT were detached. At room temperature, the plates were shaken well for 5 min by adding 500 µl of DMSO. The crystallized dye was extracted by adding 1 mL of DMSO to the well. At 570 nm absorbance, the quantity of blue dye formed was estimated. The proliferation of the cells was examined by microscopic visualization at 48X magnification. The control and drug-treated cells were treated with 10% methanol for 5 min. Then cells were viewed under an inverted microscopeto confirm the anti-proliferative efficacy of the drug.

### 2.4 Statistical analysis

The mean and standard deviation data were displayed (SD). The noteworthyvariances were calculated using a one-way analysis of variance (ANOVA). A commercial software application (SPSS version 20) was employed for statistical analysis. SPSS offers data analysis for descriptive and bivariate statistics, numerical result forecasts, and predictions for classifying groups.

## 3 Results and discussion

### 3.1 Molecular structure analysis

**FIGURE 1:**
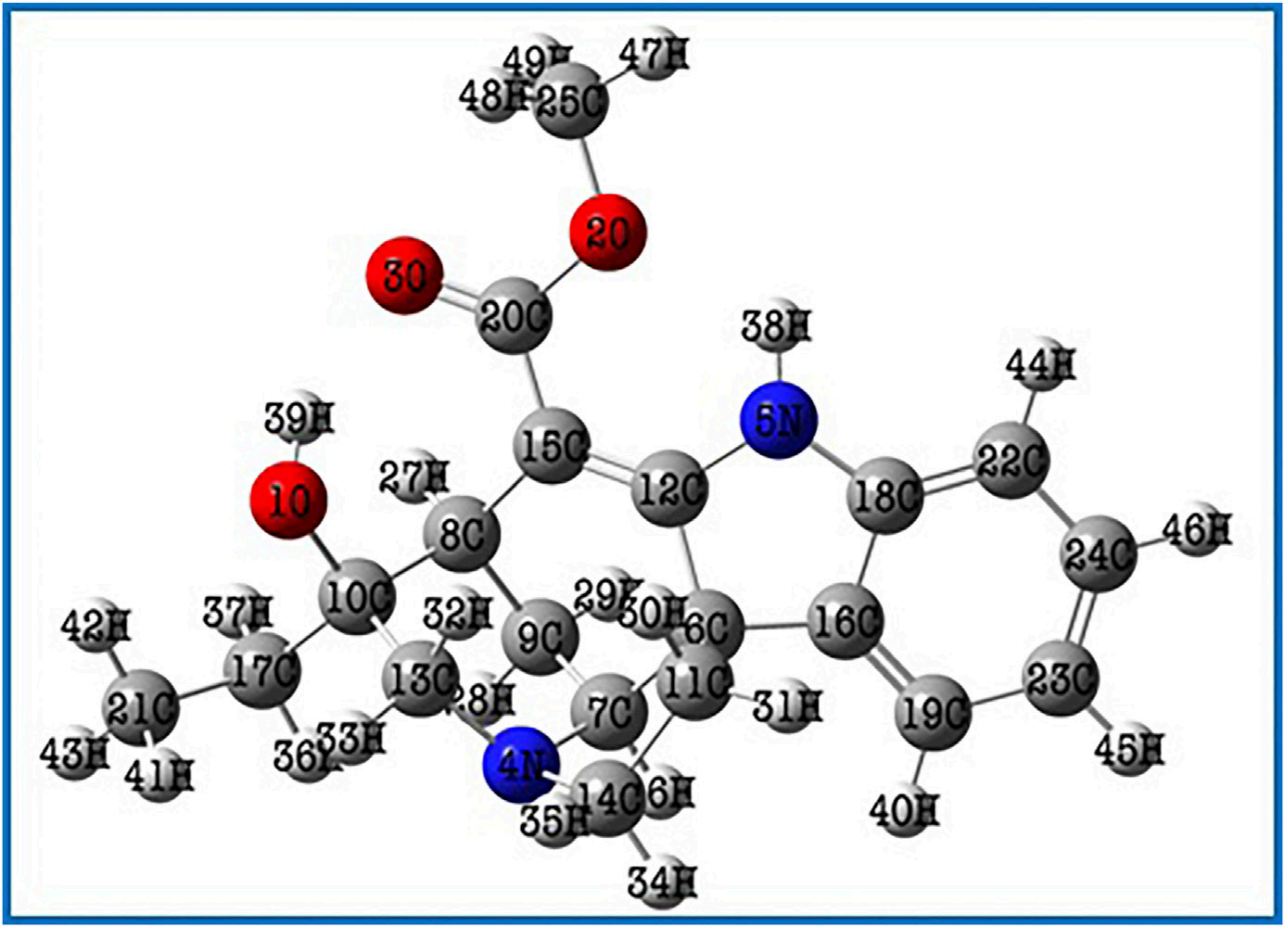
The optimized molecular structure of the Lochnericine molecule.

The Lochnericine molecule’s molecular structure was optimized using the DFT/B3LYP method with the cc-pVTZ basis set (Valarmathi et al., 2020a). Figure 1 depicts the optimized molecular structure of the lochnericine molecule, and its energy value was determined to be 1,111.43 a. u. Bond angle, bond length, and dihedral calculations collectively known as lochnericinestructural parameters were also displayed in Table 1. The C1 point group has been noticed in the molecular geometry of the lochnericine molecule. Lochnericine has been pointed to as a centrosymmetric structure due to its IR and Raman active vibrational modes. The optimized molecular structure of the Lochnericine molecule is located at a local minimum on the potential energy surface due to the absence of negative vibrational wavenumbers (Valarmathi et al., 2020b).

**TABLE 1:** The optimized structural parameters of Lochnericine molecule.

Structural parameters	Cc-pVTZ	Structural parameters	Cc-pVTZ	Structural parameters	Cc-pVTZ
Bond length Å)		Bond length Å)		Bond angle (degree)	
O1-C10	1.46	C19-H40	1.08	C7-C9-H29	109.73
O1-H39	0.98	C21-H41	1.09	C8-C9-H28	111.07
O2-C20	1.39	C21-H42	1.09	C8-C9-H29	110.09
O2-C25	1.47	C21-H43	1.09	H28-C9-H29	107.54
O3-C20	1.25	C22-C24	1.40	O1-C10-C8	109.90
N4-C7	1.49	C22-H44	1.08	O1-C10-C13	105.09
N4-C13	1.50	C23-C24	1.39	O1-C10-C17	108.86
N4-C14	1.48	C23-H45	1.08	C8-C10-C13	108.18
N5-C12	1.38	C24-H46	1.08	C8-C10-C17	111.52
N5-C18	1.41	C25-H47	1.08	C13-C10-C17	113.05
N5-H38	1.01	C25-H48	1.09	C6-C11-C14	103.66
C6-C7	1.57	C25-H49	1.09	C6-C11-H30	110.59
C6-C11	1.59	**Bond Angle (Degree)**		C6-C11-H31	110.84
C6-C12	1.53	C10-O1-H39	110.44	C14-C11-H30	111.06
C6-C16	1.52	C20-O2-C25	116.16	C14-C11-H31	112.93
C7-C9	1.52	C7-N4-C13	117.00	H30-C11-H31	107.75
C7-H26	1.09	C7-N4-C14	102.38	N5-C12-C6	106.95
C8-C9	1.54	C13-N4-C14	108.96	N5-C12-C15	128.50
C8-C10	1.55	C12-N5-C18	110.11	C6-C12-C15	124.10
C8-C15	1.53	C12-N5-H38	120.30	N4-C13-C10	114.67
C8-H27	1.09	C18-N5-H38	125.83	N4-C13-H32	111.81
C9-H28	1.09	C7-C6-C11	102.68	N4-C13-H33	107.24
C9-H29	1.09	C7-C6-C12	111.08	C10-C13-H32	105.28
C10-C13	1.53	C7-C6-C16	122.69	C10-C13-H33	110.43
C10-C17	1.54	C11-C6-C12	111.67	H32-C13-H33	107.18
C11-C14	1.55	C11-C6-C16	108.00	N4-C14-C11	104.61
C11-H30	1.09	C12-C6-C16	100.79	N4-C14-H34	109.25
C11-H31	1.09	N4-C7-C6	106.00	N4-C14-H35	110.85
C12-C15	1.37	N4-C7-C9	112.28	C11-C14-H34	110.66
C13-H32	1.09	N4-C7-H26	106.78	C11-C14-H35	113.12
C13-H33	1.09	C6-C7-C9	111.91	H34-C14-H35	108.25
C14-H34	1.09	C6-C7-H26	108.64	C8-C15-C12	119.26
C14-H35	1.09	C9-C7-H26	110.92	C8-C15-C20	116.13
C15-C20	1.44	C9-C8-C10	109.46	C12-C15-C20	123.79
C16-C18	1.41	C9-C8-C15	108.53	C6-C16-C18	107.52
C16-C19	1.38	C9-C8-H27	109.55	C6-C16-C19	132.32
C17-C21	1.53	C10-C8-C15	115.79	C18-C16-C19	119.76
C17-H36	1.09	C10-C8-H27	106.98	C10-C17-C21	113.94
C17-H37	1.10	C15-C8-H27	106.34	C10-C17-H36	109.13
C18-C22	1.39	C7-C9-C8	108.09	C10-C17-H37	108.11
C19-C23	1.40	C7-C9-H28	110.30	C21-C17-H36	109.20
C21-C17-H37	109.00	C13-N4-C7-H26	−166.63	C16-C6-C11-H30	116.61
H36-C17-H37	107.21	C14-N4-C7-C6	−41.38	C16-C6-C11-H31	−2.83
N5-C18-C16	108.93	C14-N4-C7-C9	−163.88	C7-C6-C12-N5	−155.20
N5-C18-C22	129.02	C14-N4-C7-H26	74.32	C7-C6-C12-C15	17.83
C16-C18-C22	121.99	C7-N4-C13-C10	41.81	C11-C6-C12-N5	90.80
C16-C19-C23	119.02	C7-N4-C13-H32	−77.94	C11-C6-C12-C15	−96.15
C16-C19-H40	120.87	C7-N4-C13-H33	164.83	C16-C6-C12-N5	−23.67
C23-C19-H40	120.09	C14-N4-C13-C10	157.26	C16-C6-C12-C15	149.36
O2-C20-O3	119.93	C14-N4-C13-H32	37.51	C7-C6-C16-C18	143.01
O2-C20-C15	115.03	C14-N4-C13-H33	−79.70	C7-C6-C16-C19	−44.29
O3-C20-C15	125.02	C7-N4-C14-C11	45.781	C11-C6-C16-C18	−98.09
C17-C21-H41	111.68	C7-N4-C14-H34	−72.74	C11-C6-C16-C19	74.59
C17-C21-H42	110.30	C7-N4-C14-H35	168.03	C12-C6-C16-C18	19.12
C17-C21-H43	110.37	C13-N4-C14-C11	−78.76	C12-C6-C16-C19	−168.18
H41-C21-H42	107.91	C13-N4-C14-H34	162.70	N4-C7-C9-C8	55.58
H41-C21-H43	107.44	C13-N4-C14-H35	43.48	N4-C7-C9-H28	−66.03
H42-C21-H43	109.01	C18-N5-C12-C6	20.30	N4-C7-C9-H29	175.65
C18-C22-C24	117.59	C18-N5-C12-C15	−152.32	C6-C7-C9-C8	−63.50
C18-C22-H44	121.51	H38-N5-C12-C6	179.74	C6-C7-C9-H28	174.88
C24-C22-H44	120.88	H38- N5-C12-C15	7.11	C6-C7-C9-H29	56.57
C19-C23-C24	120.46	C12-N5-C18-C16	−7.65	H26-C7-C9-C8	174.97
C19-C23-H45	119.71	C12-N5-C18-C22	170.06	H26-C7-C9-H28	53.36
C24-C23-H45	119.81	H38-N5-C18-C16	−165.68	H26-C7-C9-H29	−64.94
C22-C24-C23	121.13	H38-N5-C18-C22	12.02	C10-C8-C9-C7	−65.36
C22-C24-H46	119.16	C11-C6-C7-N4	20.63	C10-C8-C9-H28	55.77
C23-C24-H46	119.69	C11-C6-C7-C9	143.37	C10-C8-C9-H29	174.78
O2-C25-H47	104.89	C11-C6-C7-H26	−93.80	C15-C8-C9-C7	61.88
O2-C25-H48	110.06	C12-C6-C7-N4	−98.87	C15-C8-C9-H28	−176.97
O2-C25-H49	110.21	C12-C6-C7-C9	23.85	C15-C8-C9-H29	−57.96
H47-C25-H48	111.17	C12-C6-C7-H26	146.67	H27-C8-C9-C7	177.61
H47-C25-H49	111.15	C16-C6-C7-N4	142.03	H27-C8-C9-H28	−61.24
H48-C25-H49	109.27	C16-C6-C7-C9	−95.23	H27-C8-C9-H29	57.76
**Dihedral Angle (Degree)**		C16-C6-C7-H26	27.59	C9-C8-C10-O1	175.37
C25-O2-C20-O3	2.73	C7-C6-C11-C14	6.67	C9-C8-C10-C13	61.13
C25-O2-C20-C15	−176.46	C7-C6-C11-H30	−112.43	C9-C8-C10-C17	−63.79
C20-O2-C25-H47	177.04	C7-C6-C11-H31	128.11	C15-C8-C10-O1	52.33
C20-O2-C25-H48	57.37	C12-C6-C11-C14	125.77	C15-C8-C10-C13	−61.90
C20-O2-C25-H49	−63.22	C12-C6-C11-H30	6.67	C15-C8-C10-C17	173.16
C13-N4-C7-C6	77.6534	C12-C6-C11-H31	−112.78	H27-C8-C10-O1	−65.99
C13-N4-C7-C9	−44.84	C16-C6-C11-C14	−124.27	H27-C8-C10-C13	179.76
H27-C8-C10-C17	54.83	C6-C11-C14-N4	−32.14	C10-C17-C21-H41	−65.10
C9-C8-C15-C12	−22.20	C6-C11-C14-H34	85.41	C10-C17-C21-H42	54.90
C9-C8-C15-C20	147.91	C6-C11-C14-H35	−152.90	C10-C17-C21-H43	175.43
C10-C8-C15-C12	101.32	H30-C11-C14-N4	86.63	H36-C17-C21-H41	57.20
C10-C8-C15-C20	−88.56	H30-C11-C14-H34	−155.80	H36-C17-C21-H42	177.22
H27-C8-C15-C12	−139.99	H30-C11-C14-H35	−34.12	H36-C17-C21-H43	−62.24
H27-C8-C15-C20	30.11	H31-C11-C14-N4	−152.18	H37-C17-C21-H41	174.04
O1-C10-C13-N4	−165.87	H31-C11-C14-H34	−34.62	H37-C17-C21-H42	−65.93
O1-C10-C13-H32	−42.53	H31-C11-C14-H35	87.05	H37-C17-C21-H43	54.59
O1-C10-C13-H33	72.84	N5-C12-C15-C8	152.85	N5-C18-C22-C24	−176.69
C8-C10-C13-N4	−48.49	N5-C12-C15-C20	−16.45	N5-C18-C22-H44	2.67
C8-C10-C13-H32	74.83	C6-C12-C15-C8	−18.62	C16-C18-C22-C24	0.75
C8-C10-C13-H33	−169.78	C6-C12-C15-C20	172.06	C16-C18-C22-H44	−179.87
C17-C10-C13-N4	75.52	C8-C15-C20-O2	−165.54	C16-C19-C23-C24	1.35
C17-C10-C13-H32	−161.14	C8-C15-C20-O3	15.30	C16-C19-C23-H45	−178.84
C17-C10-C13-H33	−45.76	C12-C15-C20-O2	4.07	H40-C19-C23-C24	−179.03
O1-C10-C17-C21	−44.95	C12-C15-C20-O3	−175.07	H40-C19-C23-H45	0.75
O1-C10-C17-H36	−167.30	C6-C16-C18-N5	−8.39	C18-C22-C24-C23	−0.37
O1-C10-C17-H37	76.39	C6-C16-C18-C22	173.69	C18-C22-C24-H46	179.35
C8-C10-C17-C21	−166.38	C19-C16-C18-N5	177.82	H44-C22-C24-C23	−179.74
C8-C10-C17-H36	71.25	C19-C16-C18-C22	−0.08	H44-C22-C24-H46	−0.02
C8-C10-C17-H37	−45.04	C6-C16-C19-C23	−172.94	C19-C23-C24-C22	−0.67
C13-C10-C17-C21	71.44	C6-C16-C19-H40	7.45	C19-C23-C24-H46	179.60
C13-C10-C17-H36	−50.91	C18-C16-C19-C23	−0.97	H45-C23-C24-C22	179.52
C13-C10-C17-H37	−167.21	C18-C16-C19-H40	179.41	H45-C23-C24-H46	−0.19

### 3.2 Vibrational spectral analysis

**FIGURE 2:**
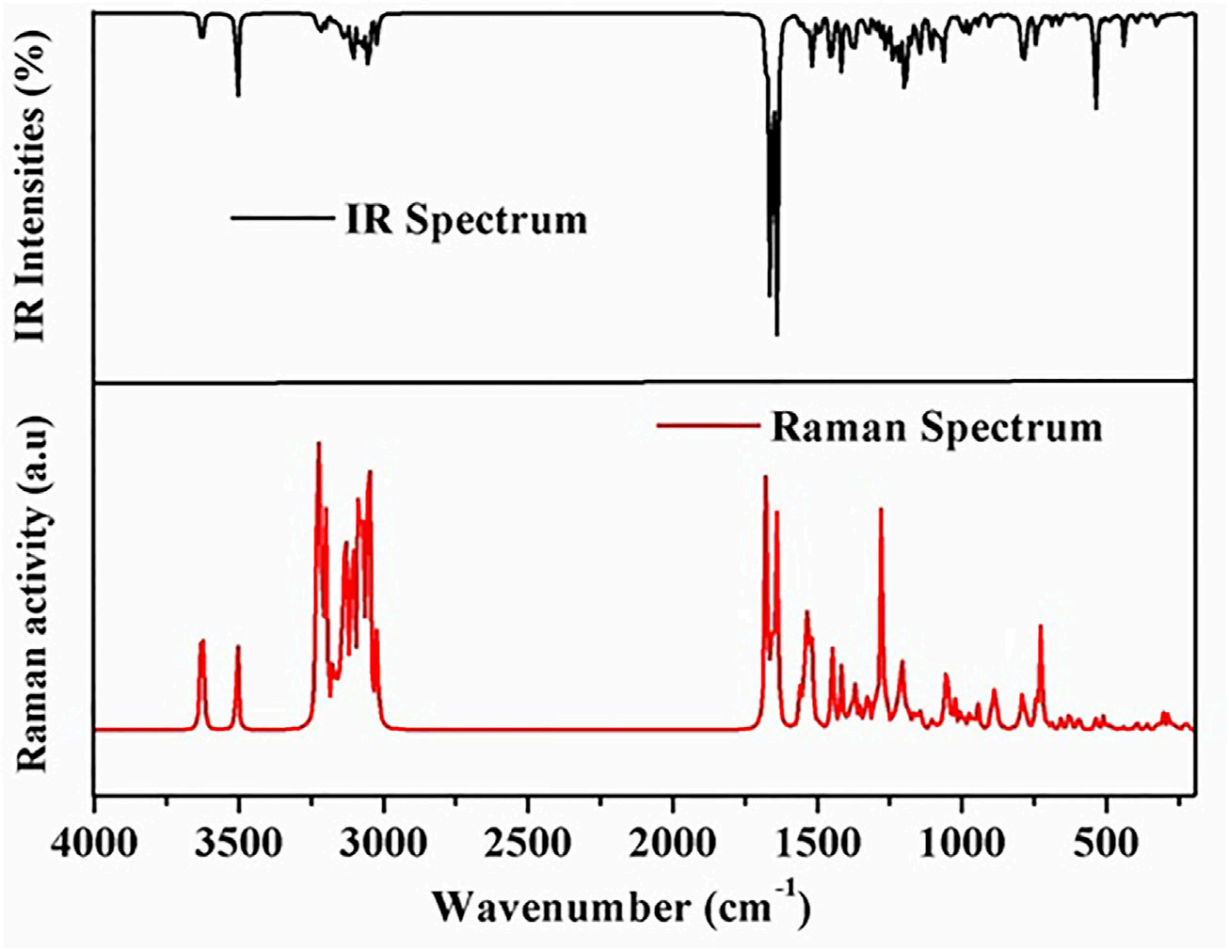
Theoretically simulated infrared and Raman spectra of the Lochnericine molecule.

49 atoms and 141 normal vibration modes are present in the lochnericine, and all belong to the same symmetry species. The calculated values oflochnericinevibrational frequencies, IR intensity, and activity of Raman scattering have been represented in Yable 2. C1 point group symmetry exists in the Lochnericine molecule. Figure 2 depicts the theoretically simulated infrared and Raman spectra of the Lochnericine bioactive compound. The experimentally determined vibrational wavenumbers and theoretically calculated vibrational wavenumber values agreed well (Areans et al., 1988; Karabacak et al., 2008; Almansouret al., 2021). The molecule’s vibrational frequencies were calculated using the VEDA 4.0 programme, which many researchers have recognized as applicable (Zhou et al., 2021).

**TABLE 2:** Theoretical IR and Raman frequencies and their assignments for Lochnericine molecule.

Mode	Wavenumber (cm^-1^)	Assignment	Mode	Wavenumber (cm^-1^)	Assignment
1	3627	ν NH (100)	41	1456	δ CH_2_ (28) + β OH (14)
2	3506	ν OH (100)	42	1450	δ CH_2_ (28) + β OH (13)
3	3227	ν CH (99)	43	1446	β CH (18)+ β NH (12)
4	3214	ν CH (98)	44	1421	ω CH_2_ (29)
5	3201	ν CH (97)	45	1416	β CH (23)+ ω CH_2_ (18)
6	3200	ν_as_ CH_3_ (97)	46	1402	β CH (22)+ ω CH_2_ (13)
7	3193	ν CH (97)	47	1392	β CH (22)+ ω CH_2_ (12)
8	3172	ν_as_ CH_3_ (95)	48	1385	β CH (38)
9	3156	ν_as_ CH_3_ (94)	49	1379	β CH (23)+ β NH (11)
10	3145	ν_as_ CH_2_ (96)	50	1377	β CH (28)+ β NH (10)
11	3135	ν_as_ CH_2_ (96)	51	1367	β CH (14)+ ω CH_2_ (26)
12	3129	ν_as_ CH_2_ (97)	52	1352	β CH (27)+ ω CH_2_ (13)
13	3113	ν_as_ CH_3_ (96)	53	1339	β CH (18)+ ω CH_2_ (28)
14	3104	ν_as_ CH_2_ (95)	54	1330	t CH_2_ (29)
15	3087	ν_s_ CH_2_ (94)	55	1322	β CH (27)+ β NH (20)
16	3085	ν_as_ CH_2_ (93)	56	1301	β CH (23)+ t CH_2_ (17)
17	3084	ν_s_ CH_3_ (94)	57	1297	t CH_2_ (32)
18	3072	ν_s_ CH_2_ (93)	58	1279	β NH (22)+ t CH_2_ (16)
19	3057	ν CH (92)	45	1262	β NH (26)+ t CH_2_ (13)
20	3053	ν CH (92)	60	1259	β NH (33)+ t CH_2_ (12)
21	3051	ν CH (93)	61	1237	β CH (29)+ β OH (22)
22	3048	ν_s_ CH_3_ (92)	62	1227	β CH (25)+ t CH_2_ (12)
23	3024	ν CH (92)	63	1217	β CH (22)+ t CH_2_ (10)
24	3021	ν_s_ CH_2_ (92)	64	1209	β CH (27)+ β OH (18)
25	1678	ν CC (50)+ β CH (32)	65	1202	β CH_3_ (36)+ β CH (14)
26	1662	ν CC (39)+ β NH (18)	66	1196	β CH_3_ (29)+ β CH (12)
27	1652	ν CC (39)+ β CH (17)+ β NH (11)	67	1183	β CH (27)+ β NH (12)
28	1640	ν C=O (87)	68	1175	β CH_3_ (23)+ β CH (11)
29	1560	δ CH_2_ (39)	69	1165	β CH_3_ (32)
30	1555	β CH_3_ (37)	70	1155	ρ CH_2_ (23)+β CH (12)
31	1544	δ CH_2_ (42)	71	1145	ρ CH_2_ (24)+β CH (11)
32	1543	δ CH_2_ (36)	72	1139	ρ CH_2_ (29)+ ω CH_2_ (22)
33	1539	β CH_3_ (39)	73	1107	ρ CH_2_ (28)+ t CH_2_ (18)
34	1537	β CH_3_ (41)	74	1099	ρ CH_2_ (19)+ t CH_2_ (14)
35	1535	δ CH_2_ (29)	75	1084	ρ CH_2_ (23)+ β CH_3_ (13)
36	1531	β CH (32)	76	1067	ρ CH_2_ (23)+ β CH_3_ (13)
37	1527	δ CH_2_ (38)	77	1059	ρ CH_2_ (29)+ β CH_3_ (11)
38	1518	β CH_3_ (46)	78	1052	Ring deformation
39	1517	β CH (38)	79	1046	ν CC (39)+ ν CO (14)
40	1492	β CH_3_ (43)	80	1026	ν CC (25)+ β CH_3_ (12)
81	1008	γ CH (42)	112	528	γ OH (23)
82	1007	γ CH (38)	113	509	ρ CH_2_ (23)+ γ OH (13)
83	996	γ CH (34)+ν CC (23)+ ν CO (11)	114	491	τ Ring
84	976	γ CH (37)	115	476	ρ CH_2_ (28)+ γ CH (18)
85	969	γ CH (29)+ β NH (11)	116	453	ρ CH_2_ (29)+ γ CH (11)
86	957	γ CH (29)	117	438	ρ CH_2_ (12)+ γ OH (23)
87	945	γ CH (39)+ν CC (21)	118	416	ρ CH_2_ (32)
88	942	γ CH (37)+ ν CC (18)	119	395	γ OH (23)
89	905	t CH_2_ (19) + β NH (10)	120	362	τCCCH (12)+ τCCCC (13)
90	894	γ CH (27)	121	355	τCCCC (13)+ τCCCN (12)
91	890	ρ CH_2_ (26)+ν CO (10)	122	328	τCCCH (13)+ τCCNO (11)
92	880	ρ CH_2_ (23)	123	321	τCCCC (13)+ τCCNO(11)
93	875	ρ CH_2_ (25) + ν CC (12)	124	307	τCCCH (12)+ τCCCC (13)
94	853	Ring Breathing	125	303	τ Ring
95	824	ρ CH_2_ (23)+ t CH_2_ (13)	126	285	τ Ring
96	804	Ring Breathing	127	267	τCCCH(12)+ τCCCC(13)
97	792	γ CH (38)	128	251	τCCCH(12)+ τCCCC(12)
98	783	γ CH (27)+ γ NH (28)	129	228	ρ CH_2_ (23)
99	776	γ CH (29)	130	219	τ CH_3_ (32)
100	745	ρ CH_2_ (16) + β NH (12)	131	179	τ CH_3_ (34)
101	740	γ NH (29)	132	153	τCCCH(12)+ τCCCC(13)
102	727	γ NH (32)	133	147	τCOCO(11)+ τCCCC(11)
103	706	Ring deformation	134	132	Ring deformation
104	687	Ring deformation	135	118	Ring deformation
105	663	Ring deformation	136	103	τ CH_3_ (23)
106	656	ρ CH_2_ (21) + β NH (13)	137	94	τ CH_3_ (12)
107	628	Ring deformation	138	77	Ring deformation
108	600	ρ CH_2_ (26) + β OH (12)	139	70	τ CH_3_ (14)
109	594	τ Ring	140	67	τCOCO(12)+ τCCCC(11)
110	582	γ CH (38)+ β CH_3_ (18)	141	55	τCOCO(12)+ τCCCC(10)
111	537	γ OH (23)			

ν, Stretching; ν_s_, symmetric stretching; ν_as_, asymmetric stretching; β, in plane bending; γ, out of plane bending; ρ, rocking; ω, Wagging; δ, Scissoring; t, twisting.

### 3.3 Frontier molecular orbitals (FMOs) analysis

**FIGURE 3:**
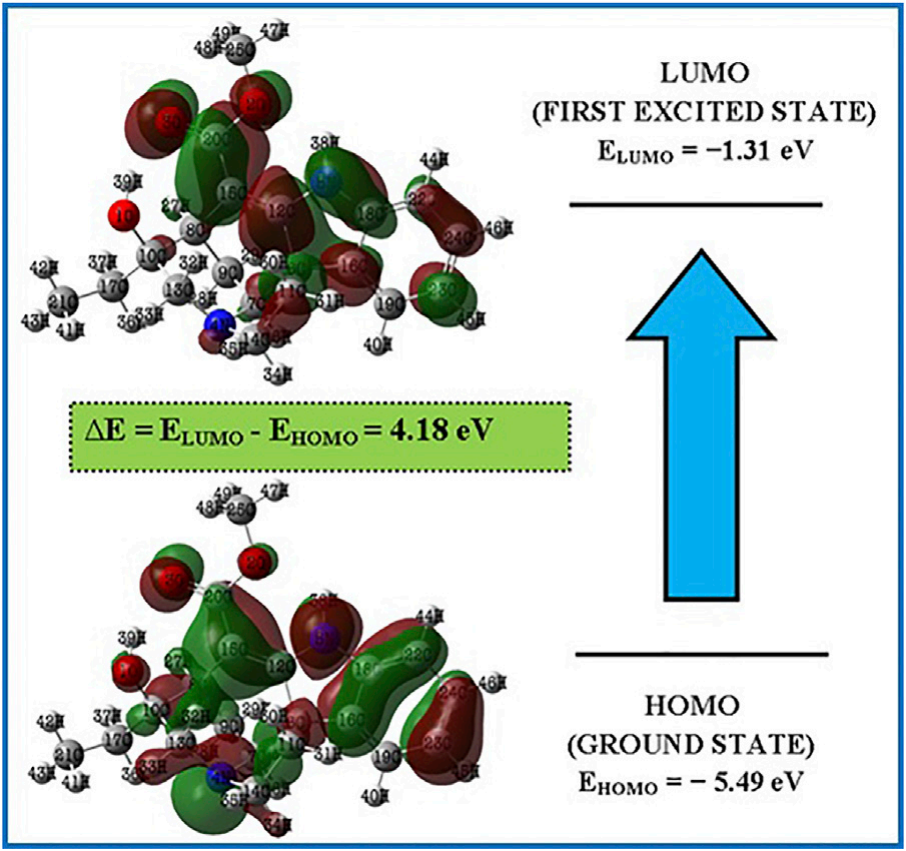
FMOs of the Lochnericine molecule.

The highest occupied molecular orbital (HOMO) and the lowest unoccupied molecular orbital (LUMO) are the two molecular orbitals that are used to analyse how a molecule interacts with other species (LUMO). The HOMO energy represents the capacity to provide electrons, whereas the LUMO energy represents the capacity to carry electrons (Koyambo-Konzapa et al., 2021). Electric and optical properties, UV-Vis spectra, and quantum chemistry all depend on FMOs. (Babu et al., 2016). The molecule’s kinetic stability and chemical reactivity—both crucial components in determining its electrical properties—are represented by the HOMO-LUMO gap. Small HOMO-LUMO gaps are linked to soft molecules that have strong chemical reactivity and limited kinetic stability. Figure 3 depicts the Lochnericine molecule’s FMOs. The FMOs’ related molecular features were calculated using Koopman’s theory (Mohamed Asath et al., 2017). Red and green are used to represent the positive and negative phases, respectively, in Figure 3. The calculated low energy gap value (4.18 eV), which also explains the intramolecular charge transfer interaction that affects the lochnericine molecule’s biological activity, supports the increased chemical reactivity of the compound (Saravanan and Blachandran, 2014). The electron affinity A) is the term used to describe the energy produced when an electron is introduced to an empty orbital, while the ionisation energy I) (5.49 eV) is used to describe the energy necessary to remove an electron from a full orbital (1.31 eV). The molecule is susceptible to electrophilic and nucleophilic reactions due to its anticipated high ionisation energy and low electron affinity. The chemical potentialμ = 3.40 eV, global softness, S = 2.09 eV, μ = 3.40 eV, global hardness, η = 0.47 eV, and global electrophilicity index, ψ = 12.29 eV, of the molecule were also computed. Based on the anticipated values of higher hardness and reduced softness, the molecule is probably stable. The molecule’s predicted chemical potential and electrophilicity index provide additional evidence that it has chemical stability comparable to that of compounds with potential bioactivity.

### 3.4 MEP surface analysis

**FIGURE 4:**
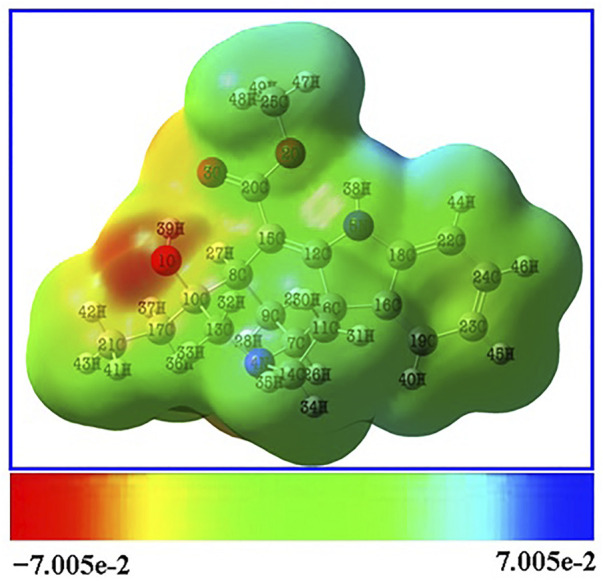
MEP surface of the Lochnericine molecule.

Red, yellow, light blue, and blue on the MEP surface (Figure 4) represent, respectively, regions that are slightly electron-rich, slightly electron-rich, slightly electron-deficient, and slightly electron-deficient. It was revealed that the oxygen atoms lone pair electrons cause the regions around them to be electron-rich (red). Moreover, the oxygen atom O1 has a stronger electronegative potential, and all of the other oxygen atoms are classified as being in the slightly electron-rich zone. The slightly electron-deficient (light blue) portion of the molecule contained all of the hydrogen atoms. The molecule’s electron-deficient (blue) portion is identified as hydrogen atom H38. The hydrogen atom H38, which is shown as being electron-poor (blue), makes up the molecule. The neutral electrostatic potential envelopes were positioned around the molecule (green). Potential electrophilic and nucleophilic attack sites are the hydrogen atom H38 and the oxygen atom O1.

### 3.5 Mulliken atomic charge distribution analysis

**FIGURE 5:**
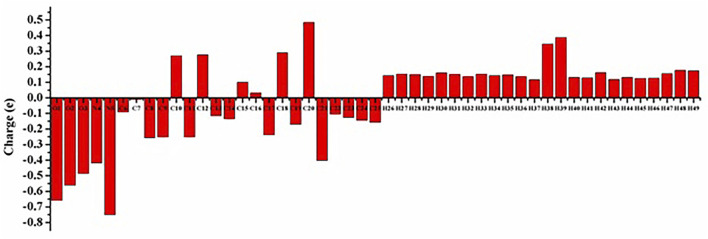
Mulliken atomic charge distribution of the Lochnericine molecule.

Mulliken’s atomic charge distribution influences the molecule’s dipole moment, electrostatic potential, electronegativity equalization, electronic structure, vibrational modes, and polarizability (Geetha et al., 2021). Because of its attachment to the two electronegative oxygen atoms, the carbon atom C20 has a higher positive charge value, as shown in Figure 5 representation of the predicted Mulliken atomic charge distribution values (O2 and O3). The highest electronegative charge values are found in the oxygen atom O1 and the nitrogen atom N5. The carbon atoms’ negative charge values significantly promote electron delocalization within the molecule.

### 3.6 Molecular docking

**FIGURE 6:**
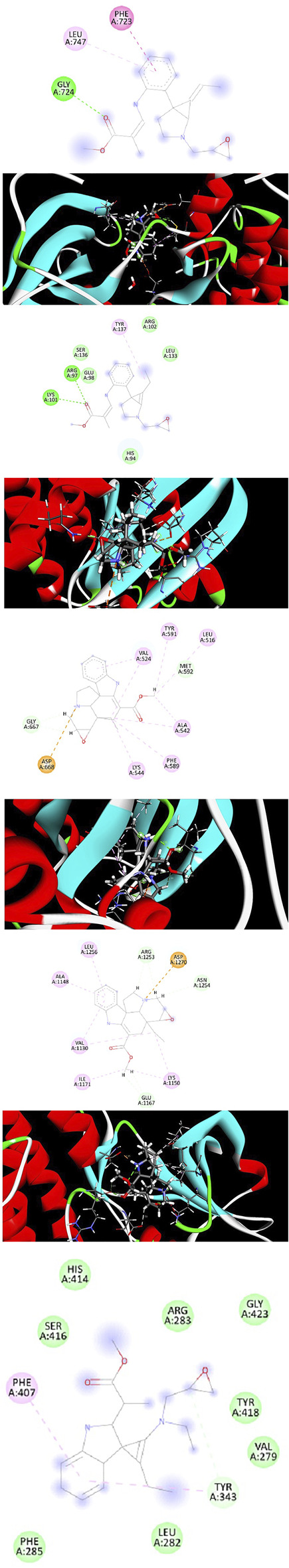
**(A)** 2d and 3d Interaction between EGFR_ Lochnericine. **(B)** 2d and 3d Interaction between KRAS_ Lochnericine. **(C)** 2d and 3d Interaction between Ntrk_ Lochnericine. **(D)** 2d and 3d Interaction between ALK_ Lochnericine. **(E)** 2d and 3d Interaction between TRIM11_ Lochnericine.

In drug designing, molecular docking is essential to determine the binding mode and efficacy of the active lead molecules. Molecular docking approaches were performed for all targeted receptor molecules. Receptor molecules docking score and interacting residues were tabulated in Table 3. EGFR receptor (PDB ID 2ITY) molecule interacts with the active lead molecules that lead to the formation of hydrogen bonds, Pi-Pi stacked, and Pi-Alkyl. Lead molecule interacts with glycine residue at the position of 724 and forms a conventional hydrogen bond. Leu 747 residue forms Pi-Alkyl interaction. Phenylalanine 723 amino acid forms the Pi-Pi Stacked. EGFR_ Lochnericine has good interaction, and thedocking score is −7.52 Kcal/Mol. 2 days and 3 days interactions between EGFR_Lochnericine were depicted in Figure 6A. A docking protocol was carried out between KRAS and Lochnericine, and the docking score between KRAS and lead molecules is −9.10 Kcal/Mol. Arginine amino acid at the position of 97 interacts with small molecules and forms the hydrogen bond. Another hydrogen bond is formed between Lysine 101 and Lochnericine. Pi-Alkyl formed by the Tyrosine 137 amino acid residues and 2 days and 3 days interactions between KRAS_Lochnericine were depicted in Figure 6B. Ntrk protein structure was downloaded from the RCSB PDB (PDB ID: 7VKO). The lead molecule with Glycine 667 amino acid residue forms the carbon-hydrogen bond. Further, Methionine 592 residue forms another carbon-hydrogen bond. Alkyl and Pi-Alkyl interactions were formed between lead molecule and amino acid residues (Valine 524, Tyrosine 591, Leucine 516, Alanine 542, Phenylalanine589 and Lysine 544). Ntrk_Lochnericine docking score is −8.24 Kcal/Mol, and the 2 days and 3 days interactions between Ntrk_Lochnericine are depicted in Figure 6C. ALK three-dimensional proteins with PDB ID:2XP2 were downloaded and prepared for docking. Alkyl and Pi-Alkyl interactions are formed by amino acid residue Leu 1,256, Ala 1,148, Val 1,130, Ile 1,171 and Lys 1,150, Glu 1,167, Asn 1,254 and Arg 1,253 amino acid residue interact with Lochnericine forms carbon-hydrogen bond. The docking score is −11.59 Kcal/Mol. 2 days and 3 days interactions between ALK_Lochnericine were depicted in Figure 6D. TRIM11 protein was modelled, and protein structure was assessed using the Saves server. TRIM11 forms Pi-Alkyl interaction with phenylalanine 407 amino acid residue. Tyrosine 343 forms a carbon-hydrogen bond, and the docking score between TRIM11_Lochnericine is −7.13 Kcal/Mol. 2 days and 3 days interactions between TRIM11_Lochnericine were depicted in Figure 6E. The docking score and interacting residues between the targeted receptor molecules and lead compound were tabulated in Table 4.

**TABLE 3:** Mulliken Atomic charges for optimized geometry.

Atom	Mulliken atomic charges	Atom	Mulliken atomic charges
O1	−0.6579	H26	0.1422
O2	−0.5613	H27	0.1517
O3	−0.4867	H28	0.1495
N4	−0.4188	H29	0.1378
N5	−0.7511	H30	0.1608
C6	−0.0910	H31	0.1503
C7	−0.0117	H32	0.1359
C8	−0.2577	H33	0.1530
C9	−0.2506	H34	0.1421
C10	0.2697	H35	0.1469
C11	−0.2508	H36	0.1354
C12	0.2759	H37	0.1159
C13	−0.1154	H38	0.3454
C14	−0.1352	H39	0.3875
C15	0.1008	H40	0.1318
C16	0.0315	H41	0.1284
C17	−0.2383	H42	0.1627
C18	0.2895	H43	0.1179
C19	−0.1709	H44	0.1311
C20	0.4833	H45	0.1241
C21	−0.4034	H46	0.1266
C22	−0.1034	H47	0.1551
C23	−0.1271	H48	0.1775
C24	−0.1444	H49	0.1734
C25	−0.1584		

**TABLE 4:** Interacting residues between receptor molecules and lead compound.

S.No	Protein_lead compound	Interacting residues	Docking score
1	EGFR_ Lochnericine	GLY_724, LEU_74 and PHE_723	−7.52 Kcal/Mol
2	KRAS_ Lochnericine	ARG_97, LYS_101 and TYR_137	−9.10 Kcal/Mol
3	Ntrk _Lochnericine	TYR_591, VAL_524, LEU_516, MET_592, ALA_542, PHE_589, LYS_544, GLY_667 and ASP_668	−8.24 Kcal/Mol
4	ALK_Lochnericine	LEU_1256,VAL_1130, ALA_1148, ILE_1171, LYS_1150, ARG_1253, ASN_1254, GLU_1167 and ASP_1270	−11.59 Kcal/Mol
5	TRIM11_Lochnericine	PHE_407 and TYR_343	−7.13 Kcal/Mol

### 3.7 ADME prediction

**FIGURE 7:**
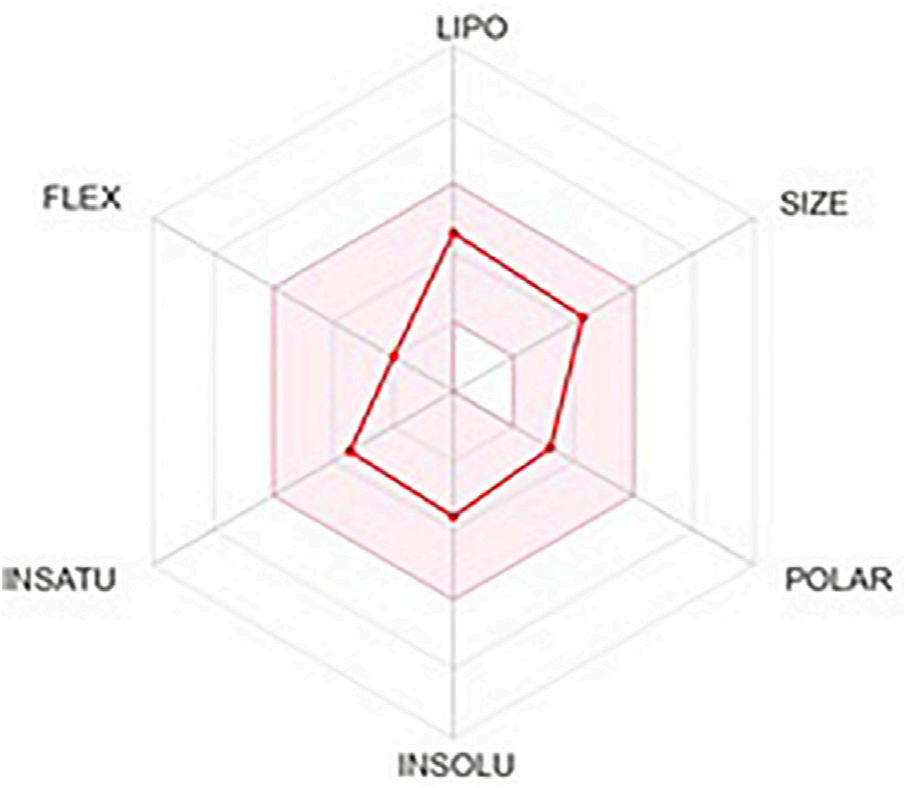
ADME prediction of Lochnericine.

In the drug-designing process, ADME prediction is essential for determining the lead molecule’s efficacy. These obey all the five rules of Lipinski. Several pharmacokinetic properties, pharmacokinetics, drug-likeness, and water solubility properties were tabulated in Table 5.Further, lead compound lipophilicity, size, insoluble, unsaturation, flexibility, and polar nature are shown in Figure 7.

**TABLE 5:** ADME prediction of Lochnericine.

ADME prediction of lochericine	
Number of rotatable bonds	3
Number of Hydrogen bond acceptors	4
Number of Hydrogen bond donor	1
Lipophilicity Log P_O/W_	3.08
Log S	−3.59
GI absorption	High
BBB permeant	Yes
Bioavailability Score	0.55

### 3.8 Molecular dynamics simulation

**FIGURE 8:**
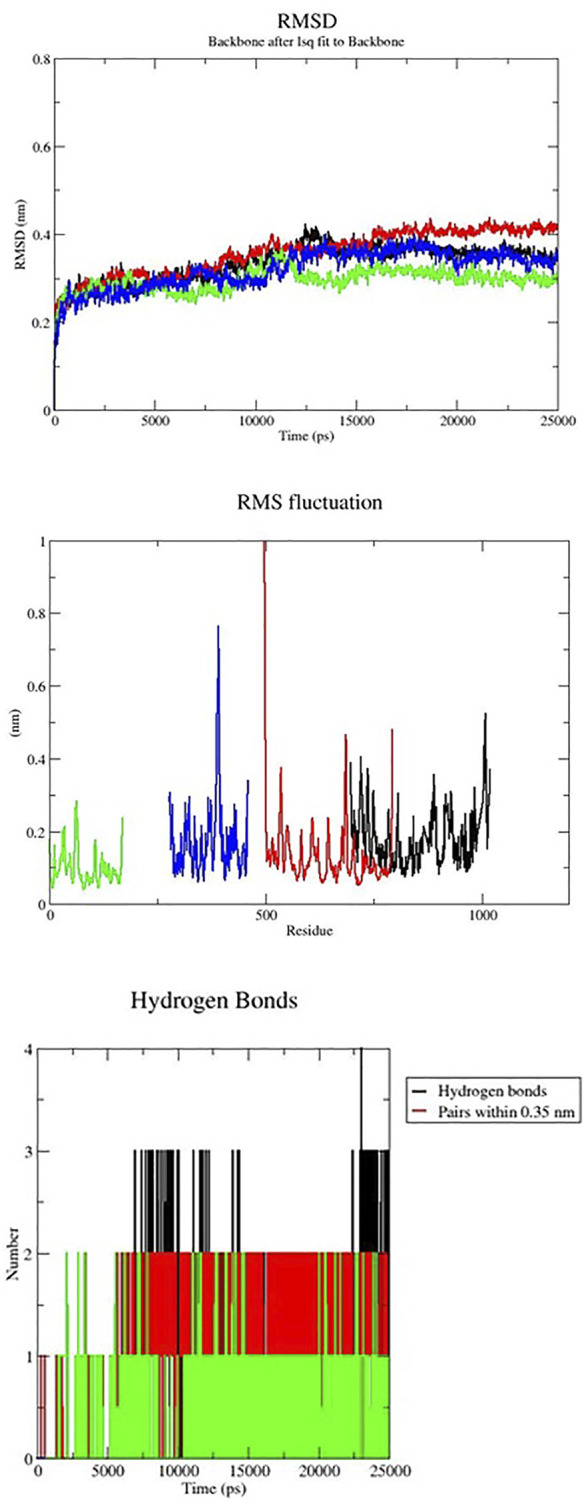
MD simulation of Nonsmall cell lung cancer targeted receptors with the lead compounds.

Gromacs package’s molecular dynamics simulations were used to examine the stability of targeted protein-ligand complexes. Based on the docking score, interaction residues, and binding affinity between the targeted proteins and the Lochnericine. For 50 ns, molecular dynamics simulations were performed. Typically, MD simulation is used to predict the behaviour of macromolecules, and it relies on classical mechanics and Newton’s equation of motion to determine the speed and position of each atom in the system under consideration. As a result, MD performs a more concise structural assessment. The difference between the initial and final structural conformational positions was determined using the RMSD (Root Mean Square Deviation). The results of MDs of protein-ligand complexes are shown in Figure 8. The average range of RMSD deviation was observed between all the protein-ligand molecules. This indicates a good RMSD deviation. All protein receptor molecules indicate favourable RMSD deviation throughout the entire simulation period. RMSF (Root Mean Square Fluctuation) assesses and computes the average deviation of a targeted protein over time from an amino acid residue reference position. The average fluctuation rate between targeted protein-ligand complexes ranges between all the protein-ligand molecules. The fluctuation between the targeted receptor molecules and Lochnericineis depicted in Figure 8. The smallest fluctuation with good stability and the fluctuation rate that occurs in the loop or disallowed region have no implications on the complexes’ stability. Hydrogen bonds in protein-ligand complexes are calculated using cut-offs for the angle between the Hydrogen Donor-Acceptor and the distance between the Donor-Acceptor. Hydrogen bond interactions between targeted proteins and Lochnericine complexes are represented in Figure 8. EGFR (PDB ID-2ITY) with the lead compound represented in the black colour. 7LGI with the active compound represented in the green colour. The red colour indicates the 7VKO protein with lochnericine. TRIM11with lochnericine represent in blue colour. Throughout the simulation runs, the GROMACS command line gmx_rmsd was used to compute the RMSD values of each targeted protein-ligand complex’s stability profile. RMSD often interprets the deviation for a set of atoms (protein, ligand, or even ligand-protein complex) from the corresponding initial reference structure. Less RMSD values would therefore be associated with considerable stability due to changes in the examined molecule’s structure. Additionally, ligands show lower RMSD values corresponding to the ligand-protein complexes. A lower RMSD value denotes higher stability for the protein-ligand complexes. All targeted receptor molecules with lead compound lesser deviation within the range of 0.4 nm. All the potential receptors of Non-small cell lung cancer protein flexibility and target Residue Root-Mean-Square Fluctuation (RMSF) for each ligand-bound protein residue root-mean-square fluctuation (RMSF) profile were calculated in order to acquire more insights into the stability of the complex binding site. The GROMACS “gmx_rmsf” command line was used to calculate the specific backbone RMSF of each protein. This flexibility validation criterion shows the contribution of specific protein residues to the structural variations of the ligand/protein complex. The RMSF calculates the average deviation for each residue from its reference position inside the reduced initial structures over time. All the targeted receptor molecules show an acceptable fluctuation range except the disallowed region. The targeted protein-ligand complex fluctuates within the range of 0.50 nm.The lowest values for the deviation below 0.40 nm and the lesser deviation below 1 nm were found while considering the examined complexes. The above RMSD and RMSF analysis shows that the targeted protein-ligand complexes have good stability and flexibility. The stability of ligand-protein complexes and the corresponding conformational changes were thought to be best understood by examining the hydrogen bond network connections between the receptors and lochnericine. It was helpful to investigate the recognized ligand-protein hydrogen bond interactions and their relative frequency using the GROMACS command line gmxhbond, which analyses the hydrogen bonds (H-bonds) between all conceivable donors D and acceptors A. EGFR receptor molecule exhibit a maximum number of 4 hydrogen bonds between the targeted receptor molecule was observed. All other receptors possess the average number of 3 hydrogen bonds between the receptor and lochnericine. All the resultant complexes show better hydrogen bond interaction throughout the simulation period. Furthermore, lochnericine induced a stabilized favoured hydrogen bond association with the targeted receptor, which was sustained for significant MD simulation. This preferential hydrogen bond pair interaction with the targeted receptors introduces the potential activity of lochnericine to block the receptor molecules of non-small-cell lung cancer.

### 3.9 Anti-proliferative estimation by MTT assay

**FIGURE 9:**
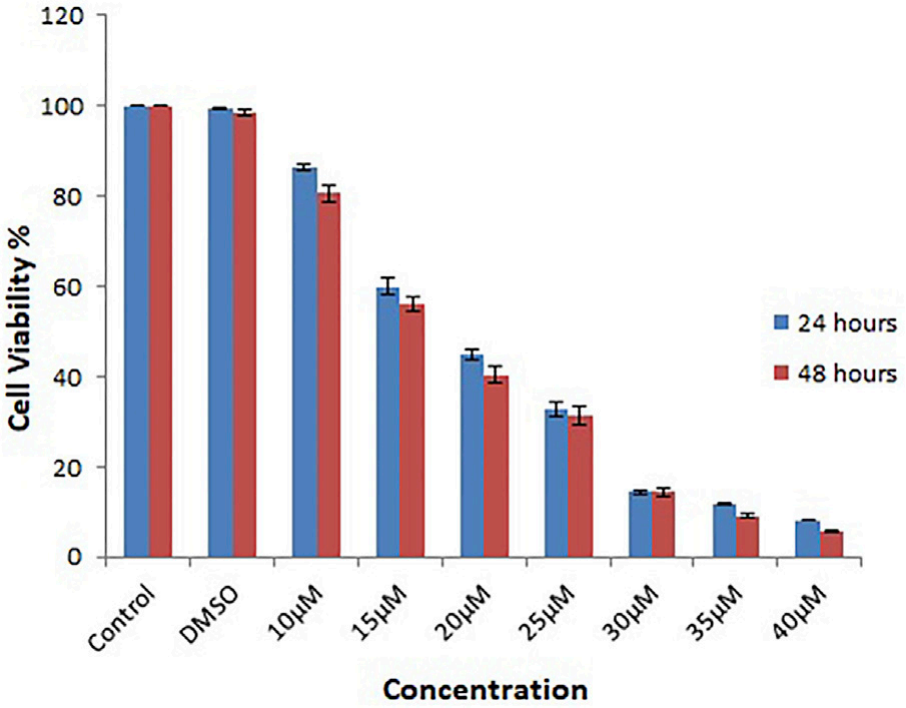
Depicts the Effect of Lochnericine on cell viability in the Human Lung Carcinoma cell line A549by MTT assay.

Several chemicals and natural compounds’ cytotoxic efficacy against malignant cells have been considered primary studies for the antitumor potential of the compounds. The conversion of MTT (3-(4, 5-dimethylthiazol-2-yl)-2, 5-diphenyltetrazolium bromide) to MTT-formation using mitochondrial enzymes in the MTT assay is a notable assay for the valuation of the proliferation of cancer cells (Sangavi et al., 2022). Treatment Lochnericine for 24 and 48 h declinedcell proliferationin a dosage and time-dependent manner, as shown in Figure 9, 10. In the present study, Lochnericinecondensed cell feasibility in a concentration-dependent manner. Lochnericine inhibition of cell growth might be linked to the induction of cell death. Thus, Lochnericinesuppressive impact on A549 cells significantly supports Lochnericine’s anti-proliferation capability.

**FIGURE 10:**
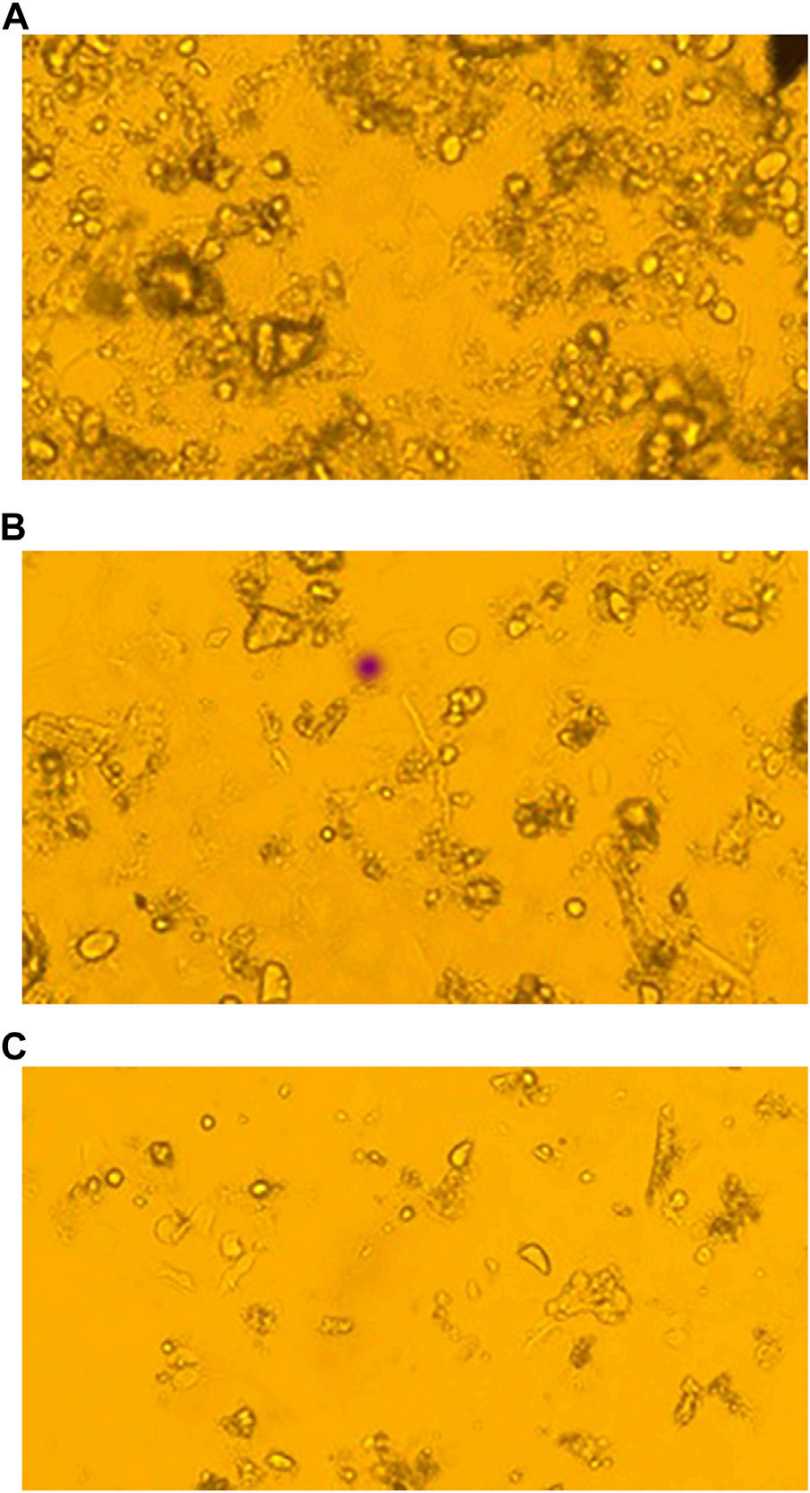
Fluorescence Microscopic images of Control and Lochnericine-treated A549 cells. **(A)** Control. **(B)** 25 µM Concentration of Lochnericine. **(C)** 30 µM Concentration of Lochnericine.

## 4 Conclusion

The optimization geometry for the lochnericine bioactive molecule was carried out using the DFT/B3LYP functional technique and the cc-PVTZ basis set. Simulated Lochnericine vibrational spectra, including infrared and Raman spectra, showed that the computed vibrational wavenumbers significantly correspond with those from earlier works of literature. The determined band gap energy value of 4.18 eV abutment lochnericine bioactivity. H38 hydrogen and O1 oxygen atom in the molecule are possible electrophilic and nucleophilic attack sites, according to a molecular electrostatic potential surface analysis. An examination of the atomic charge distribution of Mulliken verified the electron delocalization that led to the molecule’s bioactivity. non-small cell lung cancer potential targeted receptor molecules were selected and analyzed using a computational approach. During molecular docking studies, all the targeted receptor has a strong binding affinity for drugs containing lochnericine. Further targeted receptor and lochnericinedocked complexes showed good stability and lesser fluctuation during the simulation time. The protein-ligand complexes are more stable when their RMSD value is smaller. Every targeted receptor molecule has a lead compound deviation that seems to be smaller than 0.4 nm. Within a 0.50 nm range, the targeted protein-ligand complex fluctuates. While accounting for the studied complexes, the lowest values for the deviation below 0.40 nm and the lesser deviation below 1 nm were obtained. The targeted protein-ligand complexes exhibit good stability and flexibility, according to RMSD and RMSF analysis. Throughout the simulation, better hydrogen bond interaction can be seen in the resulting complexes with lochnericine.

## Data Availability

The original contributions presented in the study are included in the article/supplementary material, further inquiries can be directed to the corresponding authors.
